# Influence of central obesity in estimating maximal oxygen uptake

**DOI:** 10.6061/clinics/2016(11)02

**Published:** 2016-11

**Authors:** Christina Grüne de Souza e Silva, Barry A. Franklin, Claudio Gil Soares de Araújo

**Affiliations:** I Federal University of Rio de Janeiro, Heart Institute Edson Saad and Medical School, Rio de Janeiro/RJ, Brazil; II Exercise Medicine Clinic – CLINIMEX, Rio de Janeiro/RJ, Brazil; III William Beaumont Hospital, Preventive Cardiology and Cardiac Rehabilitation, Royal Oak, MI, USA

**Keywords:** Cardiorespiratory Fitness, Obesity, Cardiopulmonary Exercise Testing, Aerobic Fitness, Body Composition

## Abstract

**OBJECTIVE:**

To assess the influence of central obesity on the magnitude of the error of estimate of maximal oxygen uptake in maximal cycling exercise testing.

**METHOD:**

A total of 1,715 adults (68% men) between 18-91 years of age underwent cardiopulmonary exercise testing using a progressive protocol to volitional fatigue. Subjects were stratified by central obesity into three quartile ranges: Q1, Q2-3 and Q4. Maximal oxygen uptake [mL.(kg.min)^-1^] was estimated by the attained maximal workload and body weight using gender- and population-specific equations. The error of estimate [mL.(kg.min)^-1^] and percent error between measured and estimated maximal oxygen uptake values were compared among obesity quartile ranges.

**RESULTS:**

The error of estimate and percent error differed (mean ± SD) for men (Q1=1.3±3.7 and 2.0±10.4; Q2-3=0.5±3.1 and -0.5±13.0; and Q4=-0.3±2.8 and -4.5±15.8 (*p*<0.05)) and for women (Q1=1.6±3.3 and 3.6±10.2; Q2-3=0.4±2.7 and -0.4±11.8; and Q4=-0.9±2.3 and -10.0±22.7 (*p*<0.05)).

**CONCLUSION:**

Central obesity directly influences the magnitude of the error of estimate of maximal oxygen uptake and should be considered when direct expired gas analysis is unavailable.

## INTRODUCTION

There is strong evidence that cardiorespiratory fitness is inversely associated with cardiovascular and all-cause mortality 1. Cardiorespiratory fitness can be accurately assessed by directly measuring maximal oxygen consumption (VO_2_max) at peak exercise during cardiopulmonary exercise testing (CPET). However, due to the limited availability of metabolic testing resources, VO_2_max is more commonly estimated, rather than directly measured, by applying equations that take into consideration the maximal workload achieved or the exercise duration 2,3. Although measured and estimated VO_2_max are strongly associated, the error of estimate (EE) for a given subject tends to be substantial, averaging 10-20%, which far exceeds the normal error of other clinical and laboratory measurements 4.

The ability to utilize oxygen to perform work is related to an individual’s mechanical efficiency 5. Therefore, when applying a given equation to estimate VO_2_max, it is generally assumed that all subjects have the same mechanical efficiency, which is biologically incorrect and likely explains most of the EE of VO_2_max.

Among the factors that likely influence mechanical efficiency, one of the most clinically relevant is body composition, primarily due to the increased prevalence of obesity in recent decades. Obesity and, more particularly, central obesity may hinder the mobilization of the lower limbs, possibly reducing the mechanical efficiency for activities such as cycling or walking 6; therefore, there may be an increased EE of VO_2_max in overweight and obese subjects. Currently, there is very little data available regarding the role of central obesity as a modulator of the EE of VO_2_max.

Therefore, the current study was performed to determine the influence of central obesity, based on the waist to height ratio (WHtR), in the EE of VO_2_max.

## MATERIALS AND METHODS

### Sample

Our study population included volunteer adult subjects in an exercise medicine clinic who initially underwent CPET using a progressive cycle ergometer protocol between January 2008 and June 2014. All patients were evaluated for exercise prescription purposes and provided informed consent. All patients authorized the de-identified use of their collected data for research purposes. The study and retrospective data analysis were approved by the institutional ethics committee. Subjects were excluded based on the following criteria: those who had previously been tested in our clinic, those who were younger than 18 years of age, those who had undergone treadmill testing, and those who did not fulfill the criteria for a maximal CPET. An additional 100 subjects who had incomplete data were excluded. The final population sample included 1,715 subjects.

### Anthropometric measurements

Body weight was measured to the nearest 0.1 kg while the subject was barefoot and wearing light clothing. Height and waist circumference were obtained to the nearest 0.1 cm. The latter was measured in the upright position, at the umbilicus level 7, with an anthropometric tape. The WHtR, obtained by dividing waist circumference by body height, was used as an index for central obesity, as it is a more reliable and proportional measure of obesity than the waist circumference alone.

### Maximal cardiopulmonary exercise testing

Maximal CPET was conducted using an electromagnetically braked cycle ergometer (Inbrasport CG-04, Inbrasport, Brazil) and an individualized ramp protocol designed to achieve voluntary exhaustion between 8 and 12 minutes. All CPET was performed under direct medical supervision in a properly equipped laboratory. Subject seat height and body position were carefully adjusted on the cycle ergometer to provide a comfortable cycling movement. The pedaling rate was maintained between 65 and 75 revolutions per minute.

One lead CM5 or CC5 digital ECG continuous monitoring (ErgoPC Elite, Micromed, Brazil) was obtained at rest, during exercise and at 5-minute recovery periods. Resting, exercise and post-exercise measurements of heart rate (HR) (bpm) and blood pressure (mmHg) were obtained from ECG recordings and by auscultation of the right brachial artery, respectively.

During CPET, subjects expired through a mouthpiece and a pneumotachograph Prevent (MedGraphics, United States) with the nose occluded. Ventilatory analysis was accomplished using a metabolic analyzer VO2000 (MedGraphics, United States) that was calibrated daily. During exercise, expired gases were continuously collected, and the results were recorded at 10-second intervals. The data from six consecutive 10-second intervals were averaged and reported for each minute during CPET. VO_2_max represents the highest oxygen uptake value obtained, expressed as mL.(kg.min)^-1^, during CPET.

A CPET session was defined as maximum if it was not prematurely terminated due to adverse signs/symptoms and if it fulfilled physiological criteria 8. Additionally, measures of perceived exertion were obtained to assess somatic exhaustion, a score of 10 on the 0-10 Borg scale 9, which was further indicated by the inability to maintain the required pedal cadence (65-75 rev/min) despite strong verbal encouragement. The highest HR attained was not solely used as the criterion for considering a CPET session as the maximum.

### Predicting maximum VO_2_ and HR

To better characterize the study sample, VO_2_max [mL.(kg.min)^-1^] values were predicted by gender-specific equations 10: 60 - 0.55 x age (years) for men and 48 - 0.37 x age (years) for women. Maximum values of HR were age-predicted by a previously validated equation: HR max (bpm) = 208 - 0.7 x age (years) 11.

### Estimating VO_2_max

VO_2_max [mL.(kg.min)^-1^] was estimated by the gender-specific equations that were previously validated for a similar population in our exercise lab as follows: C-MEN = [maximal workload (watts)/weight (kg)] x 10.79 + 7 and C-WOMEN = [maximal workload (watts)/weight (kg)] x 9.82 + 7 12.

### Comparing measured and estimated VO_2_max

To compare measured and estimated VO_2_max, subjects were categorized by gender (1,172 men and 543 women) and then divided into three quartile ranges according to WHtR: Q1, Q2-3 (combining Q2 and Q3) and Q4. For each of these quartile ranges, VO_2_max per kg of body weight, EE (measured VO_2_max - estimated VO_2_max) in mL.(kg.min)^-1^ and percent error (%E; [(measured VO_2_max - estimated VO_2_max)/measured VO_2_max] x 100) were calculated for both men and women. Negative values of EE and %E signified that the estimated VO_2_max was higher than the measured VO_2_max, that is, the equation overestimated VO_2_max.

### Data analysis

Descriptive statistics are presented as mean and standard deviation or as percent. Demographic characteristics and CPET variables were compared by t-test or chi-square test for each gender. Pearson product-moment correlation was also calculated for measured and estimated VO_2_max in men and women. One-way ANOVA was used to compare EE and %E among the quartile ranges for each gender. Statistical analysis was performed using Prism 6 (GraphPad, USA), with 5% probability as the criterion for statistical significance.

## RESULTS

The average age of our subjects (n=1,715) was 52±15 years, with men representing 68.3% of the total sample. Considering the entire study population, 20.8% were apparently healthy, 17.2% had known coronary artery disease, and the remaining subjects exhibited diverse clinical conditions, including arterial hypertension, diabetes mellitus, obesity, pulmonary disease, or combinations thereof. Based on body mass index (BMI), 41.5% and 23.2% of the subjects were classified as overweight and obese, respectively, including 1.4% of the total sample that was morbidly obese. Regarding prescribed medications, 23.0% of the subjects were taking β-blockers, 34.9% were on angiotensin-converting enzyme inhibitors or angiotensin II receptor antagonists use, and 39.8% were using cholesterol-lowering medications.

As estimated by the WHtR, central obesity was higher in men was higher than in women (0.565±0.070 *vs.* 0.526±0.082, *p*<0.01). The WHtR results for each of the quartile ranges for men were as follows: Q1=0.378 to 0.515, Q2-3=0.515 to 0.612, and Q4=0.612 to 0.900. The WHtR results for each one of the quartile ranges for women were as follows: Q1=0.332 to 0.467, Q2-3=0.467 to 0.571, and Q4=0.572 to 0.813. Additional demographic and CPET data, with specific reference to progressive WHtR ranges, are presented in Tables 1 and 2.

### Maximal cardiopulmonary exercise testing results

**Figure 1:**
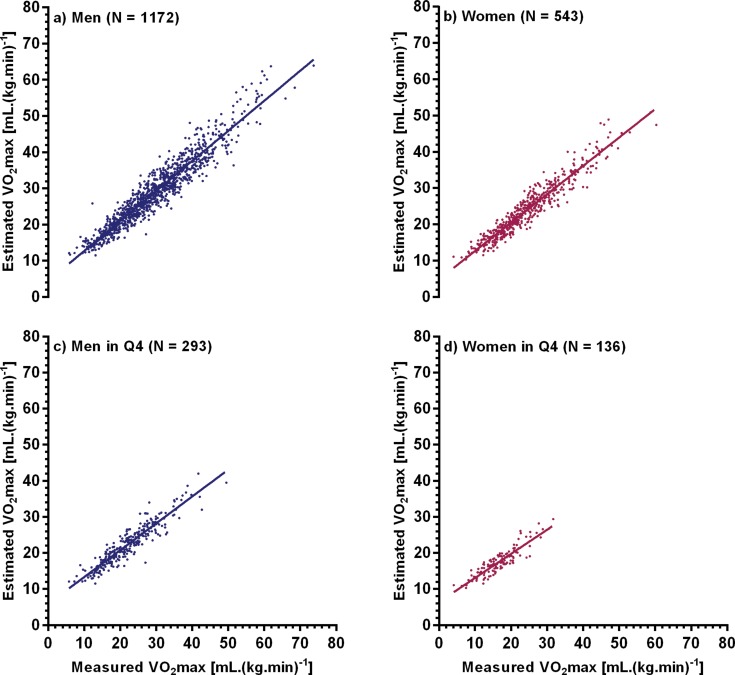
Relationship between measured and estimated VO_2_max: (a) 1,172 men, (b) 543 women, (c) men at Q4 waist-height ratio, and (d) women at Q4 waist-height ratio.

The duration of CPET averaged 10±2 minutes, with 70% of all tests lasting between 8 and 12 minutes. The maximum HR, when expressed as a percentage of the age-predicted value, was similar for men and women (92.2% *vs.* 93.0%, respectively, *p*=0.15). The maximal attained workload was higher for men than for women (172±70 *vs.* 111±45 watts, respectively, *p*<0.01). As a group, men achieved a higher VO_2_max than women (29.4±10.5 *vs.* 24.1±9.0 mL.(kg.min)^-1^, respectively, *p*<0.01). Measured VO_2_max ranged from 5.8 to 73.3 mL.(kg.min)^-1^ in men and from 4.1 to 60.1 mL.(kg.min)^-1^ in women. For both genders, these values were consistently lower for those classified in the Q4 cohort of WHtR (21.9±7.0 the expression “for men” and 16.6±5.1 mL.(kg.min)^-1^ the expression “for women”, *p*<0.01). Measured and estimated values of VO_2_max were strongly correlated in both sexes (r=0.95, *p*<0.01). For the Q4 subset, the measured *versus* estimated VO_2_max relationship remained highly significant (r=0.93 and r=0.90 for men and women, respectively) (Figure 1).

Measured VO_2_max tended to be lower than age-predicted VO_2_max, corresponding to 96.6±26.9% and 81.7±24.5% for men and women, respectively. Considering only Q4 subjects, measured VO_2_max corresponded to an even lower percent of age-predicted VO_2_max in men and women, 80.0±20.3% and 64.5±14.4%, respectively.

The values of EE were 0.5±3.2 mL.(kg.min)^-1^ for men and 0.4±2.9 mL.(kg.min)^-1^ for women (*p*=0.54). The values of %E were 0.9±13.4% for men and -1.8±15.8% for women (*p*=0.20). An analysis of %E for the three WHtR quartile ranges revealed a clear trend, with Q1 showing a slight tendency to underestimate VO_2_max and Q4 showing a strong tendency to overestimate VO_2_max for both sexes. For Q4 men, the %E was -4.5±15.8%, which was significantly larger than the %E for Q1 and Q2-3 (2.0±10.4 and -0.5±13.0%, respectively, *p*<0.01). For women, an even larger %E was identified in Q4 (-10.0±22.7%, *p*<0.01), and as observed in men, comparably lower %E values were seen in Q1 and Q2-3 (3.6±10.2 and -0.4±11.8%, respectively, *p*<0.01). When comparing EE and %E by sex in each of the three quartile ranges, no differences were observed between men and women (*p*>0.05). Nevertheless, by analyzing the magnitude of the variability of the EE and %E results, as reflected by the standard deviation of the means, it is apparent that the EE of VO_2_max remained high for any given subject, especially among those with a higher WHtR.

## DISCUSSION

Although cardiorespiratory fitness is most accurately measured during CPET 2, in most cases worldwide, VO_2_max is estimated rather than directly measured 4,13, often resulting in significant errors for any given subject. Our study addresses this issue by attempting to identify the impact, if any, of central obesity in the EE of VO_2_max. This may be especially relevant in the current era of obesity 6. While it is reasonable to estimate VO_2_max, these equations should be population-specific to account for the specific features of different populations, including body habitus.

Our study was uniquely focused on reducing the magnitude of error in estimating VO_2_max by considering separate population-specific equations for men and women. The merit of this approach was substantiated by noting that the magnitude of EE of VO_2_max was similar for men and women and that there were no other significant gender differences related to VO_2_max. After having previously developed these equations 12, in this study, we attempted to evaluate the influence of another potential modulator, central obesity, on the EE of VO_2_max. This was based on the assumption that this variable may adversely decrease mechanical efficiency and, consequently, increase the EE. In the current analysis, rather than proposing specific equations that could correct for central obesity, we focused on analyzing the consistency of these influences and potential gender differences.

When estimating VO_2_max from the peak workload achieved during maximal exercise testing, some variables may influence the EE, including body composition. First, the relationship between workload and oxygen consumption during cycling, that is, the net mechanical efficiency, has been shown to vary among lean and obese individuals. Hulens et al. 14 examined differences in exercise capacity in a large sample of lean and obese women undergoing maximal cycle ergometer testing. Net mechanical efficiency, calculated as the ratio between workload and oxygen consumed above rest to maximal exercise, was lower in women with higher relative body fat. Similarly, Lafortuna et al. 15 compared expenditure during cycling in young women of varied body habitus. Women with higher fat body mass demonstrated reduced mechanical efficiency, presumably due to the extra energy required to facilitate the associated body movement. Anton-Kuchly et al. 16 found that obese men and women exhibited a higher energy cost during submaximal exercise than their leaner counterparts, possibly due to the added energy requirements associated with muscular postural activity and/or moving the lower extremities.

Another possible explanation for our results is a difference in resting VO_2_. As previously described 12, gender-specific equations used to estimate VO_2_max assume that all individuals consume 3.5 mL of oxygen per kg of body weight per minute at rest. However, subjects with increased fat mass may have a higher absolute resting VO_2_ 17. Unfortunately, due to difficulties in establishing a standardized protocol for the accurate measurement of resting VO_2_ and, therefore, for its use in routine clinical practice 18, we were unable to quantify this variable in our study.

Previous studies have addressed other variables in obese individuals that may influence the cardiovascular response to exercise. Gondoni et al. 19 compared the HR response to progressive exercise in obese and lean individuals and reported that obese subjects had a blunted HR response and a reduced associated exercise tolerance. Fornitano et al. 20 noted differing clinical, electrocardiographic and hemodynamic responses to conventional exercise testing in morbidly obese individuals versus their overweight counterparts, reinforcing the notion that body composition should be considered when evaluating exercise performance.

This study has some methodologic features that strengthen the relevance of our findings. All CPET data were collected by four physicians in the same clinic, using the same equipment, under controlled environmental conditions and having followed standardized criteria for VO_2_max determination. The retrospective analysis was undertaken by only one investigator who was not directly involved in data collection. The sample size was considerably large and most likely reflected the clinical profiles typically seen in a medically based office practice.

There are several limitations of this study that should be acknowledged. First, age is a potential confounding variable when considering mechanical efficiency 21. Indeed, there were significant age differences among our subjects, both men and women, in the different quartile ranges, with those categorized in Q4 being significantly older. However, these subjects also demonstrated low measured VO_2_max even when adjusted for age-predicted values, which suggests that age was not the only variable that contributed to the difference in mechanical efficiency. Second, subjects were not specifically evaluated based on the use of different medications (i.e., ß-blockers), which may affect mitochondrial function 22,23, especially in the case of statins, and, in theory, the measured-estimated VO_2_max difference. Third, there are several techniques for categorizing obesity, ranging from more sophisticated approaches, such as computer tomography and DEXA, to simpler methods, such as bioimpedance or anthropometric measurements 24. Although anthropometric measurements are considered somewhat less precise, they are likely preferable and are considered reasonably accurate for rapidly evaluating the large number of subjects encountered during daily clinical practice. Accordingly, these issues should be considered in the design of future studies.

Therefore, in agreement with our previous reports 4,12,25, the application of a single equation to estimate VO_2_max may lead to modest errors in some subjects and to significant errors in others. In our study, the application of a sex-specific equation identified a distinct magnitude of error when subjects with higher and lower WHtR were compared. In subjects with higher central obesity, VO_2_max values were systematically overestimated, while the opposite occurred among subjects with less central obesity, although to a lesser degree.

Although the aforementioned studies used BMI to categorize obesity and its relationship to cardiorespiratory fitness and mechanical efficiency, it is widely recognized that BMI has numerous limitations in assessing body composition, because it fails to differentiate body fat from lean mass, and in providing indices of overall adiposity, fat distribution and metabolic risk 26-28. Moreover, BMI may be influenced by age, gender and ethnicity 26, further limiting its ability to differentiate normal weight, overweight and obese subjects.

In the present study, the WHtR provided a simple, non-invasive, anthropometric index to quantify central obesity. Moreover, unlike BMI, WHtR values are not significantly influenced by ethnic differences, age and sex, making it possible to compare central obesity among a heterogeneous cohort of men and women. Our selection of central obesity rather than overall obesity as the variable that may influence mechanical efficiency and the EE of VO_2_max was based on recent studies that have demonstrated that fat distribution has important clinical implications. Because central obesity is strongly related to visceral fat and increased cardiovascular risk 29, it seems likely that WHtR is a better prognostic discriminator than BMI 30,31,32.

In conclusion, central obesity as estimated by the WHtR influences the magnitude of the EE of VO_2_max, with a higher EE in those at the upper end of the WHtR distribution. For subjects classified in the upper WHtR quartile (men > 0.612 and women > 0.572), the use of currently available equations for estimating VO_2_max may lead to excessive errors and inaccuracies in clinical practice, especially when using functional capacity for risk stratification or in surgical clearance algorithms.

## AUTHOR CONTRIBUTIONS

All three authors contributed sufficiently to this work to take public responsibility for appropriate portions of the content. de Souza e Silva CG and Araujo CG participated in the conception and design of the study and in the acquisition, analysis and interpretation of the data. Franklin BA participated in the data analysis and critically revised the manuscript for important intellectual content.

## Figures and Tables

**Table 1:** Major characteristics and cardiopulmonary exercise testing results according to waist-height ratio ranges (expressed in quartiles) - men (N=1,172)

	Q1	Q2-3	Q4	*p* value
N	293	586	293	
**Characteristics**				
Age (years)	45±14	55±14	58±14	*p*<0.01
Weight (kg)	75.2±9.0	84.8±11.0	98.8±16.4	*p*<0.01
Height (cm)	176.7±6.5*	175.7±6.7*	173.0±7.0	*p*<0.01
Waist/height ratio	0.485±0.024	0.559±0.026	0.658±0.049	*p*<0.01
Predicted VO2max [mL.(kg.min)-1]	35.3±7.9	29.8±7.6	27.9±7.4	*p*<0.01
Predicted HRmax (bpm)	177±10	170±10	167±9	*p*<0.01
β-blocker usage (%)	15.4	25.1	37.9	*p*<0.01
**CPET Results**				
Duration (min)	11±2	10±2	9±2	*p*<0.01
HRmax (bpm)	170±20	157±25	147±27	*p*<0.01
HRmax measured/predicted (%)	96.2±9.0	92.6±11.9	87.3±13.4	*p*<0.01
Maximum workload (Watts)	208±74	170±65	141±59	*p*<0.01
Measured VO2max [mL.(kg.min)-1]	37.9±11.0	28.9±8.4	21.9±7.0	*p*<0.01
Measured/predicted VO2max (%)	109.2±28.4	98.7±24.8	80.0±20.3	*p*<0.01
Estimated VO2max [mL.(kg.min)-1]	36.6±9.9	28.4±7.2	22.2±5.6	*p*<0.01
Error of estimate (EE) [mL.(kg.min)-1]	1.3±3.7	0.5±3.1	-0.3±2.8	*p*<0.01
Percent error (%E)	2.0±10.4#	-0.5±13.0#	-4.5±15.8	*p*<0.01

* not significant between Q1 and Q2-3 (*p*=0.15);

# *p*=0.02 between Q1 and Q2-3

**Table 2:** Major characteristics and cardiopulmonary exercise testing results according to waist-height ratio ranges (expressed in quartiles) – women (N=543)

	Q1	Q2-3	Q4	*p* value
N	136	271	136	
**Characteristics**				
Age (years)	41±12	51±14	60±14	*p*<0.01
Weight (kg)	57.3±6.8	65.1±8.6	80.3±13.6	*p*<0.01
Height (cm)	165.5±6.4	162.9±6.1	159.2±5.9	*p*<0.01
Waist/height ratio	0.435±0.024	0.514±0.031	0.642±0.054	*p*<0.01
Predicted VO2max [mL.(kg.min)-1]	32.7±4.4	29.3±5.2	25.7±5.1	*p*<0.01
Predicted HRmax (bpm)	179±8	173±10	166±10	*p*<0.01
β-blocker usage (%)	8.8	14.8	28.1	*p*<0.01
**CPET Results**				
Duration (min)	11±3	9±2	7±2	*p*<0.01
HRmax (bpm)	171±17	164±21	145±26	*p*<0.01
HRmax measured/predicted (%)	95.6±7.5*	94.8±10.1*	87.0±13.1	*p*<0.01
Maximum workload (Watts)	136±47	110±42	86±34	*p*<0.01
Measured VO2max [mL.(kg.min)-1)	31.9±8.8	24.0±7.2	16.6±5.1	*p*<0.01
Measured/predicted VO2max (%)	98.0±27.2	82.1±21.1	64.5±14.4	*p*<0.01
Estimated VO2max [mL.(kg.min)-1]	30.2±7.2	23.6±6.0	17.5±3.7	*p*<0.01
Error of estimate (EE) [mL.(kg.min)-1]	1.6±3.3	0.4±2.7	-0.9±2.3	*p*<0.01
Percent error (%E)	3.6±10.2#	-0.4±11.8#	-10.0±22.7	*p*<0.01

* not significant between Q1 and Q2-3 (*p*>0.99);

# *p*=0.03 between Q1 and Q2-3; *p* values are for the comparisons among all three groups (Q1, Q2-3 and Q4) using one-way ANOVA
